# Bean leaf image dataset annotated with leaf dimensions, segmentation masks, and camera calibration

**DOI:** 10.1016/j.dib.2025.111328

**Published:** 2025-01-27

**Authors:** Karla Gabriele Florentino da Silva, Paulo Victor de Magalhães Rozatto, Kaio de Oliveira e Sousa, Lucas Dias Hudson, Artur Welerson Sott Meyer, Alemilson Fabiano Silva, Igor Tibiriçá Mendes, Alex Rodrigues Borges, Leandro Elias Morais, Luiz Maurílio da Silva Maciel, Saulo Moraes Villela, Helio Pedrini, Marcelo Bernardes Vieira

**Affiliations:** a Department of Computer Science, Federal University of Juiz de Fora, Juiz de Fora, MG, 36036-900, Brazil; b Department of Natural Sciences, Federal Institute of Education, Science and Technology of Minas Gerais, Ouro Branco, MG, 36494-018, Brazil; c Institute of Computing, University of Campinas, Campinas, SP, 13083-852, Brazil

**Keywords:** Leaf measurement, Deep learning, Semantic segmentation, Fiducial marker, Area estimation

## Abstract

Leaf dimensioning is relevant for analyzing plant responses to several conditions such as soil fertility, availability of light, agricultural pesticide effect, and access to water in the soil or periods of drought. In this paper, we present a dataset composed of 6981 images of 612 common bean leaves (*Phaseolus vulgaris*). We captured the images of each leaf accompanied by a fiducial marker and annotated the known leaf dimensions (area, perimeter, length, and width). We provide annotations concerning image segmentation, known area uniformly distributed over the leaf region, real area of the marker region, marker pose, capture conditions, and camera calibration. This dataset can be useful for developing deep learning algorithms for leaf dimensioning and related problems. Therefore, there is a potential to contribute to computer vision and plant physiology researchers and specialists.

Specifications Table

**Table:** 

Subject	Artificial Intelligence, Computer Vision and Pattern Recognition, Agronomy and Crop Science
Specific subject area	Deep learning and Machine learning, leaf dimensions estimation
Type of data	Images (.jpg), Annotations (.xml, .raw).
Data collection	We collected images from 612 bean leaves accompanied by a fiducial marker using a smartphone camera (Galaxy M31, SM-M315F). After removing the leaves from the plant, we drew and cut the contours on a paper sheet. From these contours, we measured the width and length using a ruler and the perimeter through a curvimeter. The contours were weighed to determine the leaf area based on the paper grammage. We annotated the marker and leaf masks of each image by using annotation packages (Photoshop, Supervisely, and our tool). Additionally, we estimated the camera matrices and marker localization for each image from the library for ArUco marker detection. We revised the annotations to avoid mistakes and improved the data quality.
Data source location	The images were collected in the city of Ouro Branco, Minas Gerais, Latitude −20.535912, Longitude −43.711031, Brazil.
Data accessibility	Repository name: Leaf on Stem Image Dataset Beans (LSID-Beans) Data identification number: 10.17632/f42hwwrpgn.2 Direct URL to data: https://data.mendeley.com/datasets/f42hwwrpgn/2

## 1 Value of the Data

• The dataset images are useful for developing deep learning methods for non-destructive leaf dimension estimation. We provide each leaf's known area, perimeter, width, and length, which can be used to train supervised machine learning algorithms.
• Methods developed using the dataset can help to monitor plants and crops at the beginning of the phenological phase. This monitoring is relevant for plant physiology specialists since the morphological leaf parameters are helpful to explain various processes, such as climate change, ecological relationships, and agricultural productivity.
• Detailed annotations of the segmentation masks allow for training and validating methods to segment the objects of interest on the images. The images present natural backgrounds in various environmental conditions, allowing enough variability for generalization.
• Each one of the 612 leaves has around 11 images in different positions with approximated camera calibration matrices. This is useful for pose estimation and stereoscopic reconstruction. The dataset provides 3D marker poses, which might be used as a reference for the scene.
• At the limit of our knowledge, no other datasets contain images of leaves in the natural environment, with annotations of real dimensions. The diversity of annotations (area maps, segmentation masks, camera matrix, and marker pose) makes this dataset suitable for several applications.
• The proposed methods for leaf measurement using the proposed dataset can be adapted for leaves of other species. Specifically, other dicotyledonous plants such as soybean, which are important commodities for the world economy. They present phenotypic features similar to beans. Also, our dataset can be complemented with other leaf image datasets for application in problems such as: leaf segmentation, leaf diseases detection and identification, species classification, and identification of bean species.

## 2 Background

Leaf morphological parameters (area, perimeter, width, and length) are useful for analyzing plant responses to conditions such as soil fertility [1], fruit quality [2], availability of light [3], agricultural pesticide effect [4] and access to water in the soil or periods of drought [5]. Therefore, methods for measuring leaf surface parameters based on image analysis can contribute to research in plant physiology. In this context, a dataset containing images with annotated known leaf dimensions can be applied to train deep learning supervised algorithms. We propose a dataset of bean leaves, including information on known area, perimeter, width, and length. A fiducial marker with a known dimension accompanies the leaves on each image. Additionally, we provide segmentation masks, which allow us to train algorithms to identify the objects in the scene. The dataset also includes the 3D position and orientation of the marker for each image. This 3D information contributes to a better analysis of the scene. We chose leaves of common beans (*Phaseolus vulgaris* L.), a dicotyledonous plant whose morphology is similar to soybean foliage, one of the most relevant commodities for the world economy [6].

## 3 Data Description

The Leaf on Stem Image Dataset Beans (LSID-Beans) contains 6981 images of 612 leaves. The number of images per leaf varies from 6 to 16. The resolution of the images is 512 × 512 pixels. We organized our data into one folder by image, named as the leaf number, from “001” to “612”. Since each plant has two leaves, pairs of consecutive leaves belong to the same plant (for example, leaves “001” and “002” belong to the first plant). Inside the folder of each leaf, we have four subfolders: “images,” “annotation,” “segmentation,” and “area.” The folder “images” contains the images of the leaf in the JPG (Joint Photographic Experts Group) format, named in the format “leaf_N_M.jpg,” where N is the number of the leaf, and M is the number of the image.

**Fig. 1:**
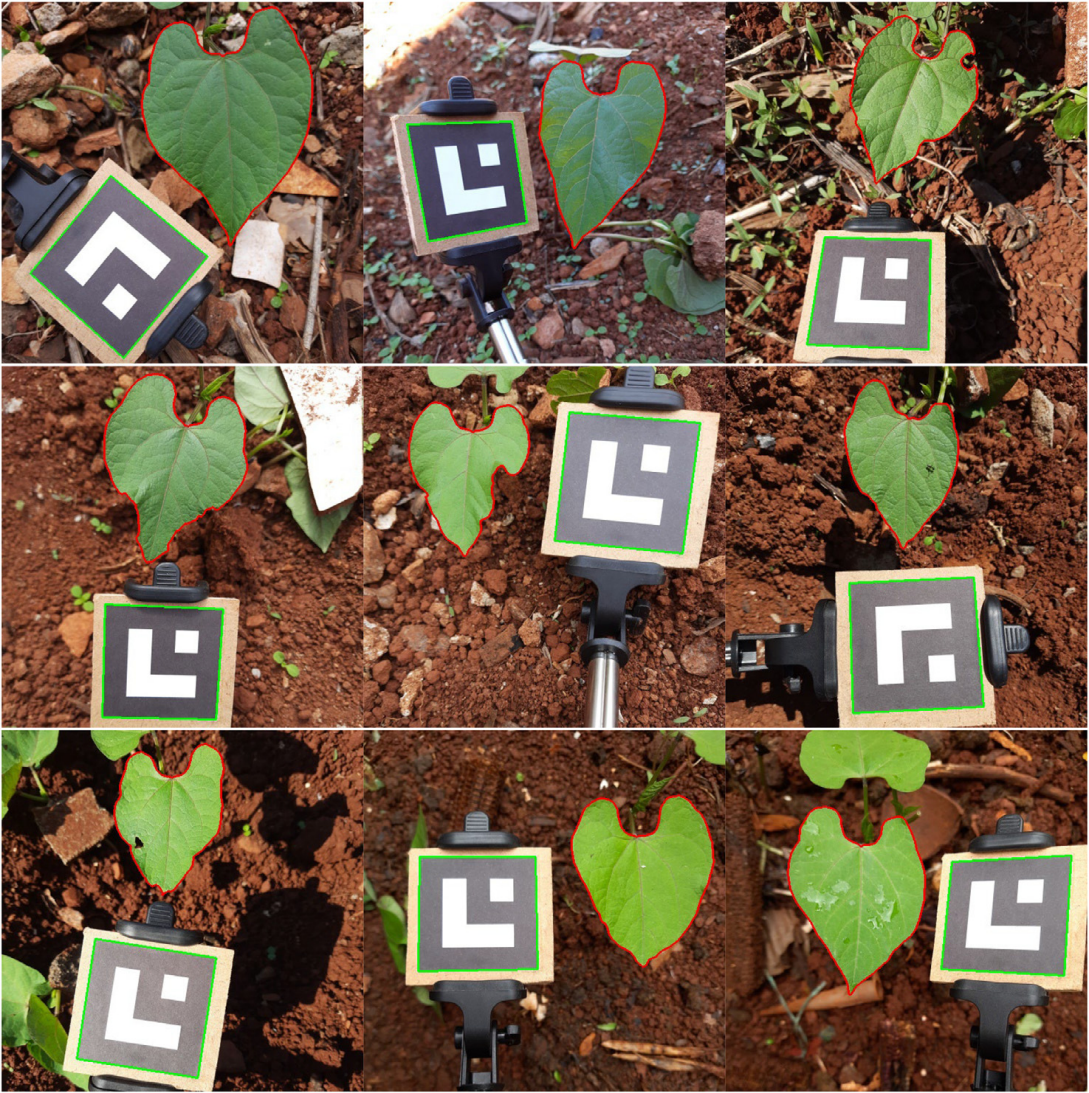
Examples of dataset images and annotation of leaves and markers. The contours of the leaves are highlighted in red and the markers in green.

The “segmentation” folder contains RAW files representing the segmentation mask of each image. Each file stores a 512 × 512 matrix of integers, where each value indicates the class of the respective pixel. The possible values are 0 (background), 1 (leaf), and 2 (marker). Each file is named as “leaf_N_M_segmentation.raw.” Section **Image Annotation** describes the process of annotating the segmentation masks. Fig. 1 presents some images of the dataset and the contours annotations.

In the “area” folder, there are RAW files comprising area maps of each image of the respective leaf. Each RAW file stores a 512 × 512 matrix of real numbers (floats) for three regions of interest: leaf region, marker region and background. Each pixel of the leaf region has the same value: the known leaf total area over the total number of pixels of the leaf region. Notice that the area is constant for all pixels of the leaf region. Furnishing the real area of each pixel of the leaf with high accuracy and precision requires expensive approaches and is outside the scope of this work. However, our dataset contains the calibration matrices that can be used for multi-image stereoscopy to approximate the leaf depth map. Nevertheless, the marker is a planar object with controlled dimensions. Consequently, each pixel of the marker region has an accurate estimation of its real area. The pixels of the background are set to zero because our setup does not provide enough information for its estimation. We have multiplied all values of the RAW file by 1000 to allow the storage using single precision float numbers. Each file is named as “leaf_N_M_area.raw.” Section **Area maps** describes the procedure to perform the pixel area annotation.

The folder “annotation” contains an XML (Extensible Markup Language) for each image. Each file describes information about the image and the captured scene. Table 1 presents the main tags used in the file. Each file is named as “leaf_N_M_annotation.xml.”

**Table 1:** Description of the annotation tags.

Tag	Description	Type
<image-name>	Name of the image.	Text
<observation>	Observation about the capture conditions, such as illumination, eating holes on the leaf, water on the leaf surface, etc.	Text
<image-size> (subtags 〈height〉, 〈width〉, <depth>)	Image dimensions.	Integer
<leaf-number>	Number of the leaf.	Integer
<marker-real-area>	Real area of the marker in square centimeters.	Real
<marker-projected-area>	Total area on the 3D marker plane computed from the projection of all marker's pixels.	Real
<normalized-corners>	A list of points as tags 〈x1〉, 〈y1〉, 〈x2〉, 〈y2〉, 〈x3〉, 〈y3〉, 〈x4〉, 〈y4〉 representing the position of each marker corner, normalized by the image dimension.	Real
<leaf-area>	Known leaf's area in square centimeters.	Real
<leaf-width>	Known leaf's width in centimeters.	Real
<leaf-length>	Known leaf's length in centimeters.	Real
<leaf-perimeter>	Known leaf's perimeter in centimeters.	Real
<bbox>	Bounding box of the leaf, composed by the tags 〈x〉 and 〈y〉 representing the upper-left corner and the tags 〈width〉 and 〈height〉 indicating the bounding box dimensions. All values are normalized by the image dimensions.	Real
<normalized-polygon>	A list of points as tags 〈x1〉, 〈y1〉, 〈x2〉, 〈y2〉, …, 〈xn〉, 〈yn〉 representing the position of the points of the leaf contour, normalized by the image dimension.	Real
<camera-matrix>	The intrinsic matrix of the camera.	Real
<tvec>	The 3D position of the marker in the camera coordinate system (in centimeters).	Real
<rvec>	The rotation vector of the marker in the camera coordinate system.	Real
<rotation-matrix>	The 3 × 3 rotation matrix obtained from <rvec>	Real
<extrinsic-homogeneous-matrix>	The extrinsic homogeneous 4 × 4 matrix of the marker.	Real
<model-projection-matrix>	The model projection 3 × 4 matrix of the marker.	Real

**Fig. 2:**
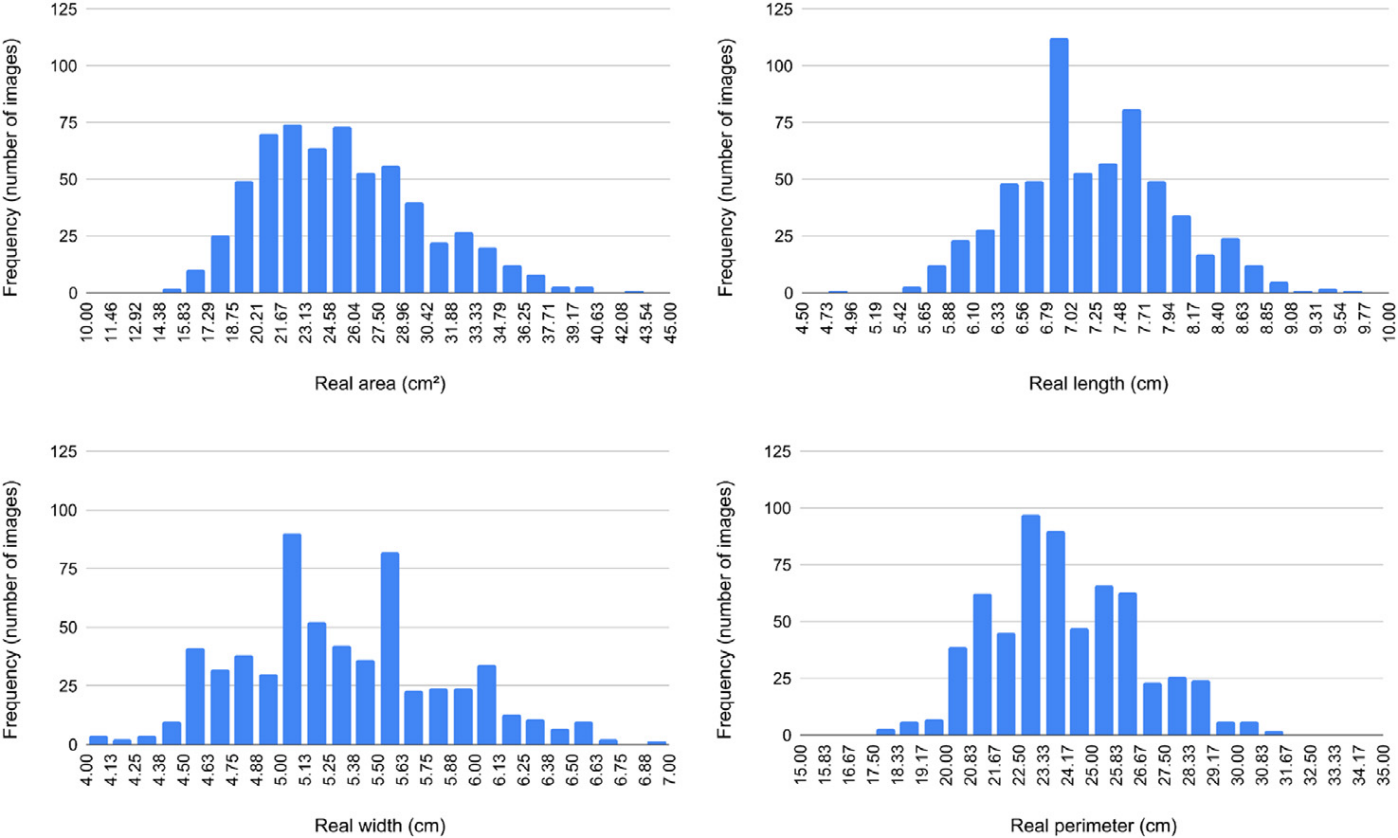
Distribution of the images in the dataset according to the known leaf dimensions.

Fig. 2 shows the distribution of images in the dataset by known area, width, length, and perimeter of the leaf. We propose a 5–5-fold cross-validation for our dataset. For each cross-validation, we split the dataset into 5 folds. We kept the images of the leaves of the same plant in the same fold to avoid bias in the learning processes. Additionally, we have distributed the leaves in the folds to maintain a similar area distribution as the entire dataset.

We provide a folder named “cross-validation” containing 5 subfolders named “cv_1,” “cv_2,” “cv_3,” “cv_4,” and “cv_5” for describing the cross-validations. In each folder “cv_i,” we include 5 subfolders named “split_1,” “split_2,” “split_3,” “split_4,” and “split_5.” Finally, the folders “split_i” contain two CSV files named “train” and “test,” indicating the images of the respective training and test sets for that split. Section **Cross-validation protocol definition** details our proposed cross-validation protocol.

## 4 Experimental Design, Materials and Methods

**Fig. 3:**
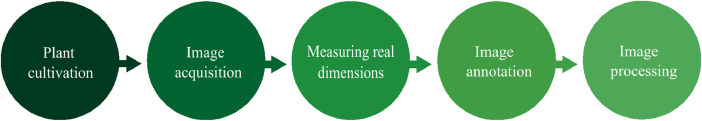
Steps of the dataset construction.

Fig. 3 shows the steps performed to build our dataset. We describe each step in the next sections. The source codes used to process the data are available in this repository: https://github.com/gcg-ufjf/LSID-Beans-Scripts.

### 4.1 Plant cultivation

**Fig. 4:**
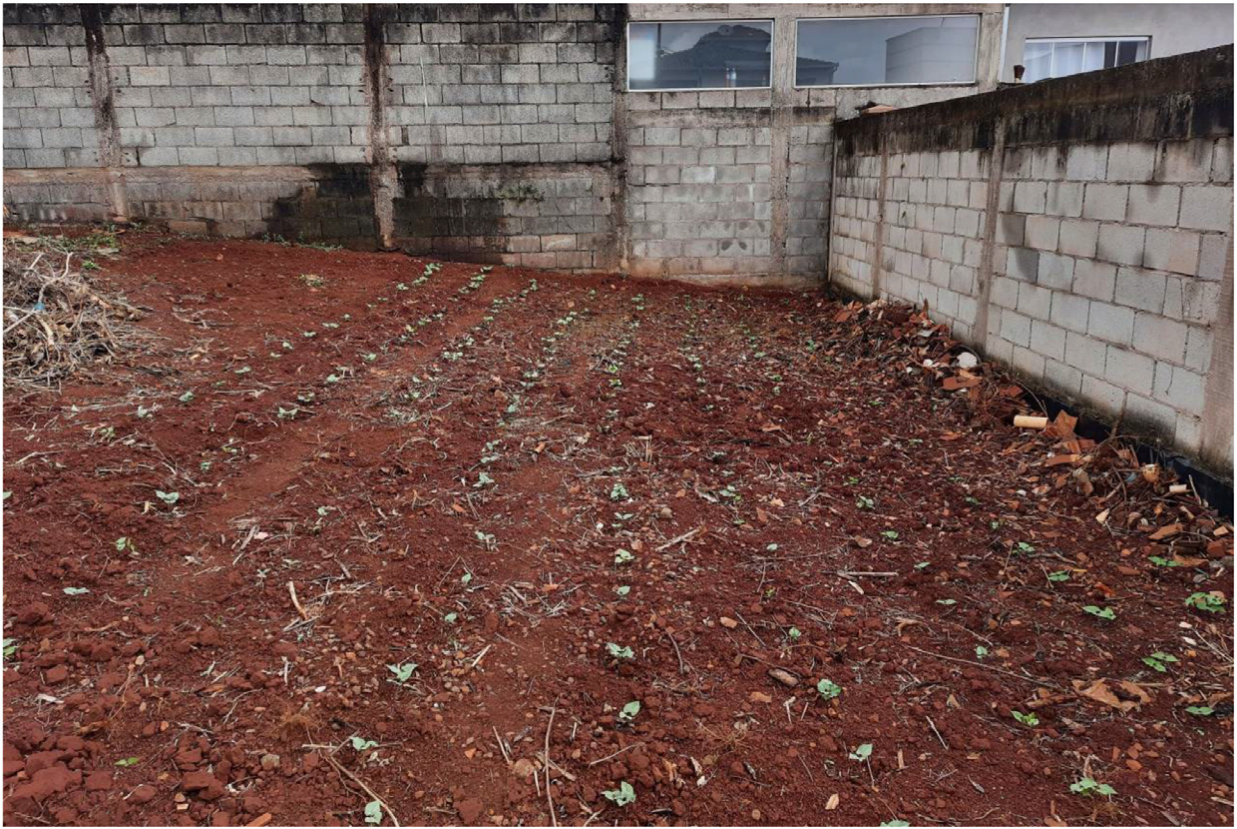
Layout of plants on the ground.

We selected black bean seeds and carried out planting in April 2022. On average, 3 seeds were sown in each pit, made with the aid of a hoe, along 9 rows of 30 plants. The soil used had never been cultivated and had rejects of construction material on the side walls. Subsequently, this fact contributed positively to a variation of the background of the scene according to the position of the plant. Fig. 4 shows the layout of the plants on site. We obtained 306 plants (612 leaves) for image acquisition, as described in the next section.

### 4.2 Image acquisition

We captured the images at the stage V2 of the plant development. At this stage, the primary leaves are fully expanded in the horizontal position [7]. This characteristic allows the capture of images of the leaf surfaces in the perpendicular direction of the camera. Additionally, in stage V2 there are many pest attacks, both in soil and leaves, which reduce the leaf area. In addition, there is greater vulnerability to water stress, reducing the leaf area growth over time. The occurrence of these problems at the beginning of cultivation can trigger irreversible damage to the common bean plants. Therefore, from a physiological point of view, it is relevant to monitor the culture throughout this phase of development.

We adopted a fiducial marker of the ArUco library as a scale and perspective reference. Developed by Garrido et al. [8], these markers are widely used in virtual and augmented reality applications and present good detection under non-uniform lighting conditions. One of the main advantages of this library is the possibility of creating configurable dictionaries. Customizing the marker size and number of bits per application is possible. In this way, the library treats only specific markers, reducing computing time.

We defined a dictionary containing only one marker of 3 × 3 size consisting of bits equal to [[1,0,1], [1,0,0], [1,1,1]] and standard edge width (equivalent to one internal bit). We chose a combination of bits that presented well-defined lines and angles. The custom marker was printed with dimensions 5 cm x 5 cm (25 cm^2^) on adhesive paper and subsequently affixed to an MDF (Medium Density Fiberboard) board of similar size. To facilitate the positioning of the marker in the scene, we used a selfie stick as support.

The capture device was a smartphone model Galaxy M31 (SM-M315F). The camera was used in Pro Mode, with manual focus adjustment, and the remaining settings (ISO value, shooting speed, exposure, white control, and color tone) with automatic adjustment. Thus, we fixed the focal length at 5.23 mm with an aperture of f/1.8. The original JPG images' resolution was 3468 × 4624 pixels (64-megapixels) and a 3:4 aspect ratio.

For camera calibration, we applied the Zhang algorithm [9], which obtains intrinsic and extrinsic parameters of the camera from views of a pattern of known actual dimensions. We used 15 images of a chess board with 9 × 6 squares of 15 mm printed and affixed to a glass plate. We captured the images by moving the device at different angles, approximately 20 cm away from the plane of the board. The calibration estimates the camera matrix:

$$M_{cam}=\left[\begin{array}{ccc} f_{x} & 0 & c_{x}\\ 0 & f_{y} & c_{y}\\ 0 & 0 & 1 \end{array}\right],$$

where $f_{x}$ and $f_{y}$ are the focal lengths, $c_{x}$ and $c_{y}$ are the coordinates of the optical center projection.

For the image acquisition, we positioned the smartphone perpendicular to the plane of the objects of interest (leaf and marker) at approximately 20 cm. We observed the limits of the camera focus so that only the extension of one leaf (other leaves with occlusion) was visible in the scene. In addition, we tried to ensure that the planar marker and the leaf were approximately coplanar in the scene, considering the average plane of the leaf. Thus, we changed the position of the marker so that at least one of its sides remained the same height as the leaf. In the case of all the images of a leaf, we tried to maintain a balance between the number of poses with greater and smaller inclinations of the marker. Fig. 1 shows examples of the arrangement of objects in the scene. Notice that the marker appears in different perspectives around the leaf (bottom, left, and right).

### 4.3 Measurement of real dimensions

**Fig. 5:**
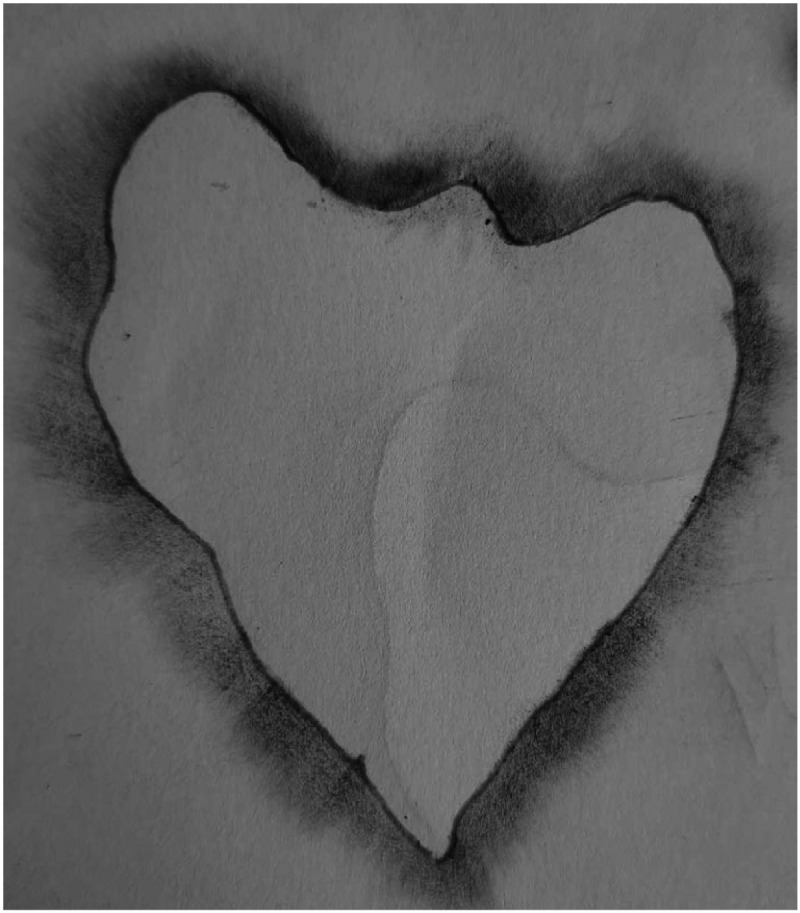
Extraction of the leaf contour.

After the capture, the two leaves of each plant received identification numbers. Subsequently, we removed each leaf from the plant and immediately drew the contour on a sheet of A4 paper to avoid a loss of mass by dehydration. For contour extraction, due to the fragility of bean leaves, we first used a powder pigment and then strengthened the shape with a graphite pencil (Fig. 5). We disregarded eating holes inside the surface. Thus, we considered only the longest perimeter contour in these particular cases.

Each contour was adequately cut and used to obtain the leaves' known dimensions (in centimeters). For the area, we used a method described by Pandey and Singh [10] based on the weight of the paper. For this, we have got the weight p of the contour using a precision balance. A specialized technician performed this step in the Laboratory of Nature Sciences of the Federal Institute of Education, Science and Technology of Minas Gerais (IFMG) - Campus Ouro Branco. Finally, we obtained the area a through the formula:

$$a=\frac{p}{g},$$

where g is the grammage of the paper. We used a curvimeter to estimate the leaf perimeter. This device is used to measure routes containing curves on maps and charts using a micro-sprocket connected to a pointer. We performed successive measurements of the same contour to obtain better precision until the same perimeter value was obtained two consecutive times. The width and length, in turn, were obtained with the aid of a ruler.

### 4.4 Image annotation

In the annotation step, we obtained two masks for each image, one for segmentation and another for the area. Initially, we accurately identified the leaf and marker in each image. Fig. 4 shows examples of the polygons generated for segmentation purposes. Due to the greater complexity of the leaves, a specific procedure was employed for their annotation, as described in the Subsection **Leaf annotation**. Subsection **Marker annotation** presents the steps employed to extract the marker's region. The algorithms mentioned in this section are implementations of the OpenCV library [11].

### 4.5 Leaf annotation

**Fig. 6:**
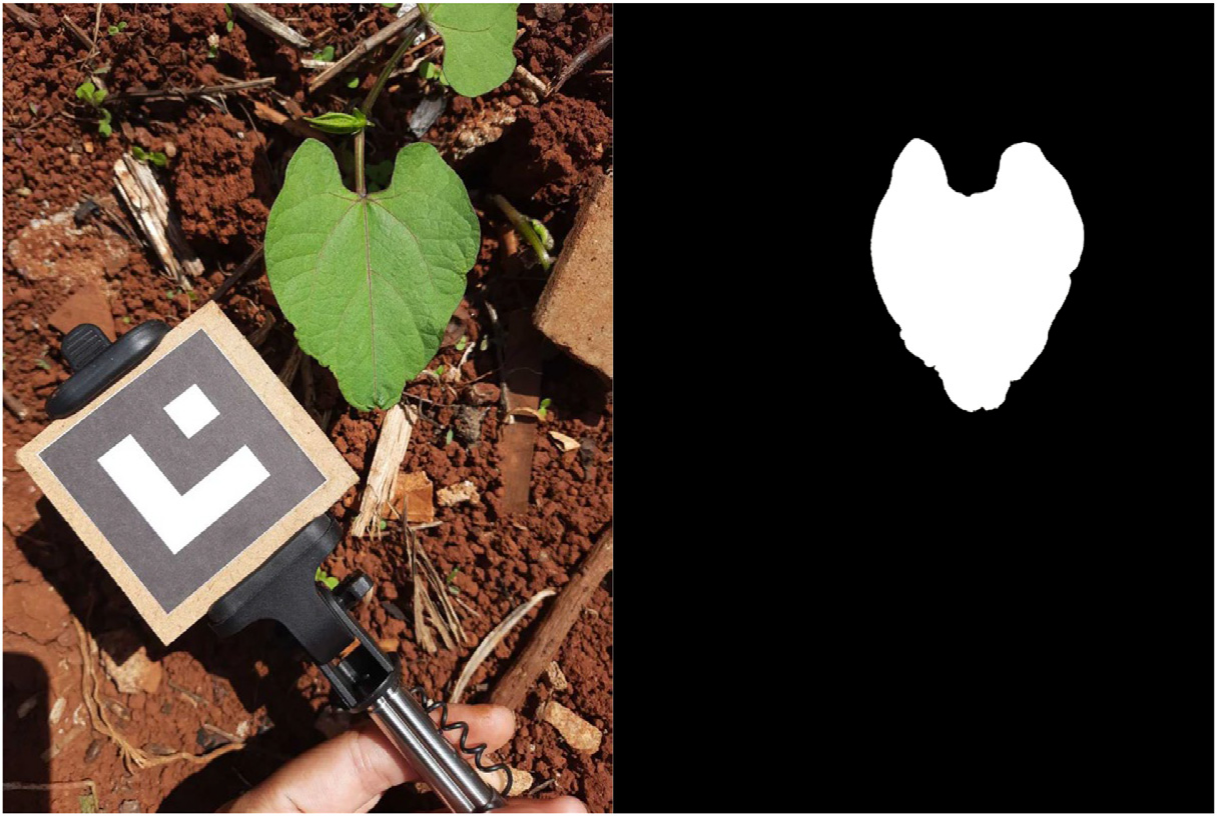
Comparison between the original image (left) and the initial generated binary mask (right).

The leaf's region presents an irregular shape with challenging angles. Therefore, the polygon annotation was the most appropriate, allowing a more precise and flexible delimitation of its outline. The first step to extract the contour was to create an initial segmentation mask using the “Quick Selection Tool” available in Adobe Photoshop® 2023 (Adobe Systems Incorporated, San Jose, CA, USA). This procedure was essential to facilitate the separation of the region of interest (leaf) from the rest of the scene. At the end of this process, we obtained a binary image $I_{b}$, where the leaf is white, and the background is black (Fig. 6).

Next, for the initial vectorization of the leaf contour, we applied the algorithm of Suzuki and Abe [12] in the image $I_{b}$. This algorithm extracts a set C:

$$C\;=\;\left\{C_{1},\;C_{2},\;\cdots ,\;C_{n}\right\},$$

where each $C_{i}$ is a contour, represented by the points that compose it:

$$C_{i}=\;\left\{\left(x_{1},\;y_{1}\right),\left(x_{2},\;y_{2}\right),\;\cdots ,\;\left(x_{m},\;y_{m}\right)\right\},$$

where each $\left(x_{j},\;y_{j}\right)$ is the position of a point (pixel) on the image that composes the contour. If |C|>1, we should discard contours, for example, internal clippings of the leaf. Then, we performed a filtering based on the area in pixels of each contour, calculated by using Green's formula [13]. Thus, we obtained a set of areas:

$$A\;=\;\left\{a_{1},\;a_{2},\;\cdots ,\;a_{n}\right\},$$

where each $a_{i}$ is the area of the respective contour $C_{i}\in C.$

We considered that the contour $C_{l}\;$ with the largest area in A represents the entire leaf. To minimize the number of points in $C_{l}$, we performed a polygonal approximation by applying the Douglas-Peucker algorithm [14], generating a new set $C_{l}^{p}\subseteq C_{l}$.

**Fig. 7:**
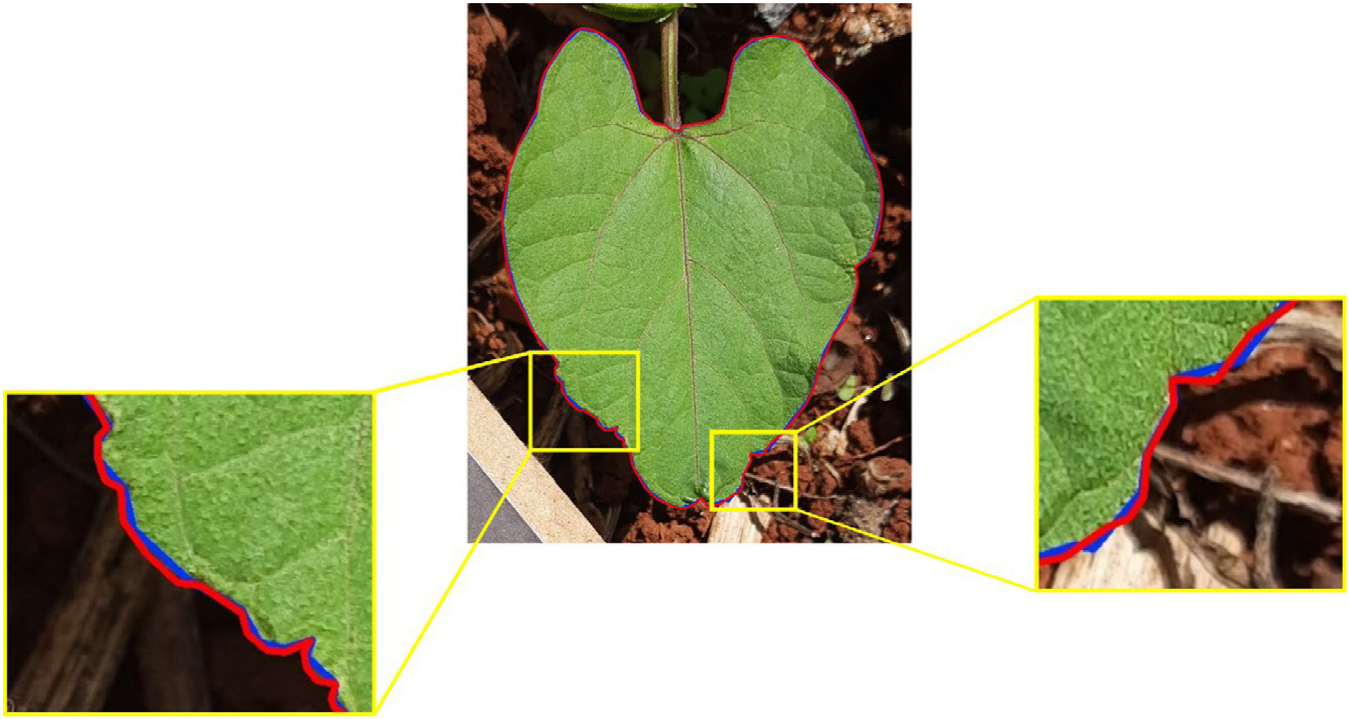
Comparison between the initial segmentation of the leaf (blue) and the revised contour (red). Highlights show parts of the leaf with more irregular curvatures.

Finally, we performed a manual refinement of the polygon using the “Basic Image Labeling Toolbox” tool available on the Supervisely [15] platform. As an alternative, we developed a new tool to boost the annotation step, which is available online [16]. The points of the set $C_{l}^{p}$ were adjusted and repositioned as needed, while new points were added to cover gaps along the edge. At the end of this stage, we conducted a rigorous review of the consistency of the annotations, drawing the polygons on the original images. Fig. 7 illustrates a comparison between the contour extracted from the mask generated by Photoshop (blue) and the revised contour (red).

### 4.6 Marker annotation

**Fig. 8:**
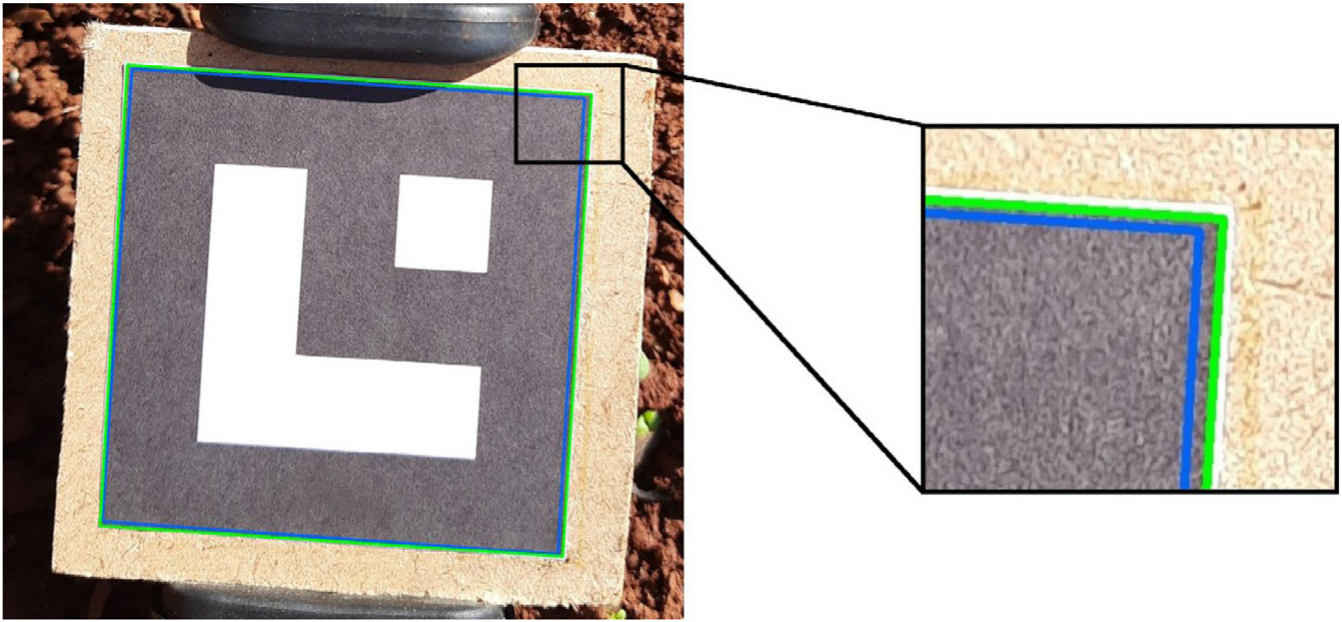
Comparison between the initial detection of the marker (blue) and the revised polygon (green). The highlight shows that the corner detected by ArUco was offset from the expected.

Since the marker presents a simple plane geometry, extracting its four corner points is enough to represent it. Initially, we applied the algorithm of Garrido et al. [8] implemented by the ArUco module, which detects the markers present in an image based on the predefined dictionary. Thus, we obtained a set:

$$M\;=\;\left\{\left(x_{1},\;y_{1}\right),\;\left(x_{2},\;y_{2}\right),\;\left(x_{3},\;y_{3}\right),\;\left(x_{4},\;y_{4}\right)\right\},$$

where each $\left(x_{j},\;y_{j}\right)$ represents the position of a marker's corner on the image. Subsequently, we exported this set and revised it using the annotation tools described in Subsection **Leaf annotation**. We drew the polygon formed by the points of the set M on the original image for visual analysis. Fig. 8 compares the marker detected by ArUco and the revised marker.

Subsequently, we applied the algorithm of Collins and Bartoli [17] to obtain the vectors of translation t and rotation r of the marker. This algorithm uses the corners of the marker along with the camera matrix and the distortion coefficients obtained from camera calibration to estimate the marker pose. Then, we applied Rodrigues' formula to obtain the rotation matrix R (3×3):

$$R=\cos\left(\theta \right)I+\left(1-\cos\left(\theta \right)rr^{T}-\sin\left(\theta \right)\right)\left[\begin{array}{ccc} 0 & -r_{z} & r_{y}\\ r_{z} & 0 & -r_{x}\\ -r_{y} & r_{x} & 0 \end{array}\right],$$

where θ is the norm of r and I is the matrix identity. Thus, the homogeneous extrinsic matrix $M_{ext}$ (4×4) is:

$$M_{ext}=\left[\begin{array}{cccc} r_{11} & r_{12} & r_{13} & t_{x}\\ r_{21} & r_{22} & r_{23} & t_{y}\\ r_{31} & r_{32} & r_{33} & t_{z}\\ 0 & 0 & 0 & 1 \end{array}\right],$$

where the values of $r_{11}\;$ to $r_{33}\;$ come from matrix R, and $t_{x}$, $t_{y}$, $t_{z}\;$ from vector t. The projection matrix of the model $M_{proj}\;$(3×4) is obtained by multiplying the camera matrix by the homogeneous matrix:

$$M_{proj}=\left[\begin{array}{ccc} f_{x} & 0 & c_{x}\\ 0 & f_{y} & c_{y}\\ 0 & 0 & 1 \end{array}\right]\left[\begin{array}{cccc} r_{11} & r_{12} & r_{13} & t_{x}\\ r_{21} & r_{22} & r_{23} & t_{y}\\ r_{31} & r_{32} & r_{33} & t_{z} \end{array}\right].$$

All markers’ polygons were manually revised in the proposed dataset. They were normalized in the original image before the image cropping and resampling.

### 4.7 Image processing

**Fig. 9:**
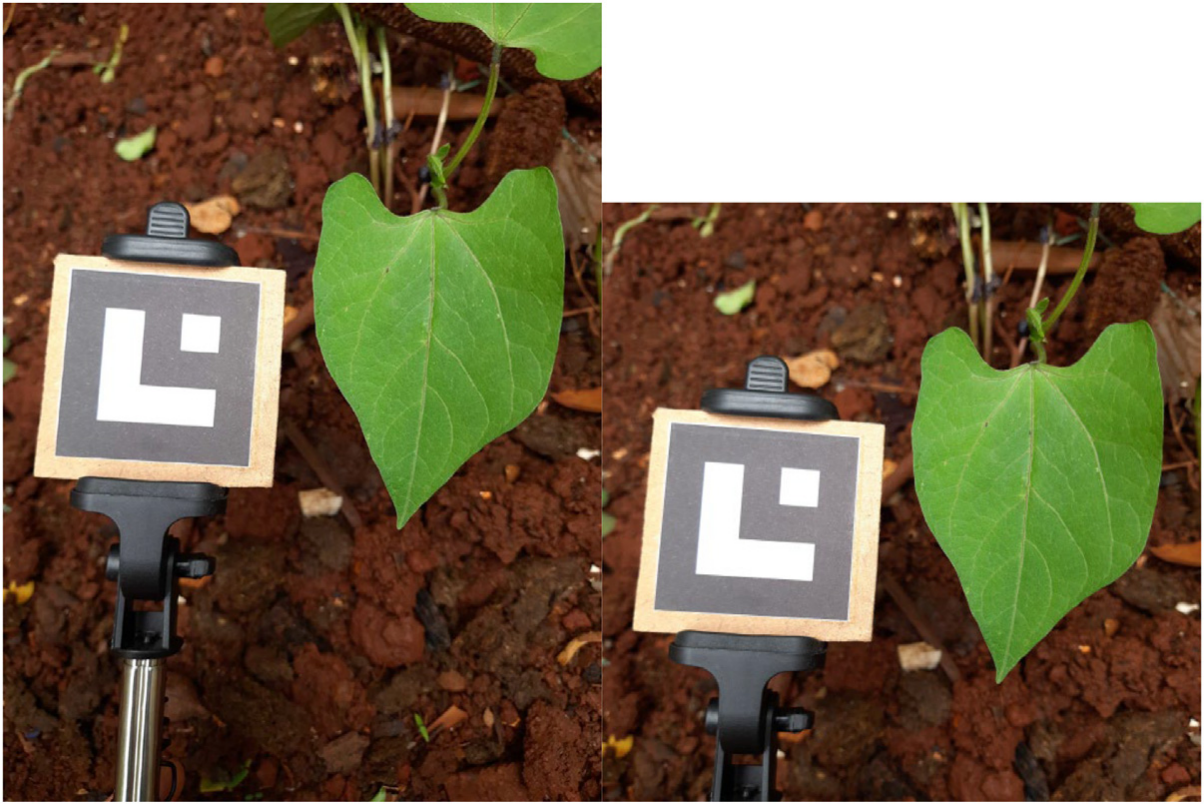
Comparison between an original image (left) and the extracted square image (right).

The original images were captured in high resolution, with 3468 × 4624 pixels. However, the training of neural networks usually employs smaller inputs due to the high consumption of computational resources. In addition, the original files are also considerably large (around 4 MB). Therefore, we have resized the images to a manageable dimension. Before that, an image with 1:1 aspect ratio containing the leaf and marker was cropped from the original image as vertically centralized as possible. This crop is important to match typical inputs of neural networks and eliminate less relevant information from the scene background. The vertical offset for the cropped image $I_{redu}$ was computed from a bounding box containing the leaf and marker contours (having coordinates in the original image $I_{orig}$) such that both objects were entirely preserved. Original images having bounding boxes whose heights are greater than the original image's width were discarded, since this means that the leaf and marker cannot appear entirely in an image with 1:1 aspect ratio without padding. Next, we cropped the image and reduced the resolution by applying a sampling by area to avoid aliasing effects. A new image of 512 × 512 pixels is obtained at the end of the process, whose all annotated coordinates and calibration matrices are accordingly adjusted. Fig. 9 shows an example of the cropping process.

The subsection **Segmentation masks** describes the process of obtaining the masks for segmentation, and Subsection **Area maps** presents the steps performed to construct the area maps for the leaf and marker.

### 4.8 Segmentation masks

**Fig. 10:**
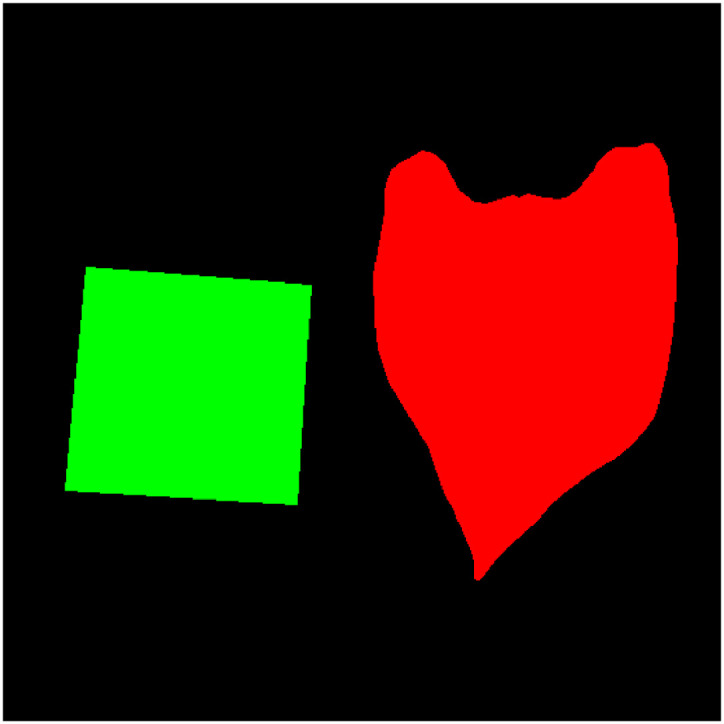
Example of the segmentation mask. Black, red, and green pixels represent the background, leaf, and marker, respectively.

We generated segmentation masks by labeling the pixels of the images in three classes: background (0), leaf (1), or marker (2), as illustrated in Fig. 10. For this, we have used the polygons $C_{l}$ and M annotated for leaf and marker identification, as described in Section **Image annotation**. Each point of the contour was transported from $I_{orig}$ to $I_{redu}$, by the affine transformation:

$$\left[\begin{array}{c} x^{\prime }\\ y^{\prime }\\ 1 \end{array}\right]=\left[\begin{array}{ccc} r & 0 & 0\\ 0 & r & o_{y}\\ 0 & 0 & 1 \end{array}\right]\left[\begin{array}{c} x\\ y\\ 1 \end{array}\right],$$

where (x,y) is the point or pixel in the original image, r is the ratio between the original and reduced resolution, $o_{y}$ is the height of the new origin, and $\left(x^{\prime },\;y^{\prime }\right)$ is the pixel resulting in the reduced image. Finally, the segmentation mask can be obtained by drawing closed polygons formed by the points of $C_{l}$ and M, according to each label, on a black image.

### 4.9 Area maps

For the area estimation task, we proposed a matrix in which each position contains the size of the pixel, estimated based on the expected area of the objects (Section **Measurement of real dimensions**). The background is not considered for measuring and all of its positions are set to zero.

All points of the leaf region are set to the same area value, i.e., all pixels have a constant average area. The pixel size is the ratio between the leaf's total area (cm^2^) and the total number of pixels of that region in the image. No procedure was applied to estimate the leaf geometry in this dataset version.

Before defining the values of the maker pixels, we needed to work around the ArUco pose ambiguity problem in some images. We set the pixel area of the marker by considering its three-dimensional reconstruction from ArUco. We projected the four vertices of each pixel on the plane associated with the marker in the three-dimensional space. Thus, given a pixel (x,y) belonging to the marker's region, the coordinates of each vertex can be obtained as follows:

$$\begin{array}{c} v_{1}=\left(x-0.5,\;y-0.5\right),\\ v_{2}=\left(x-0.5,\;y+0.5\right),\\ v_{3}=\left(x+0.5,\;y+0.5\right),\\ v_{4}=\left(x+0.5,\;y-0.5\right), \end{array}$$

where $v_{1}$, $v_{2}$, $v_{3}$ and $v_{4}$ refer to the upper-left, lower-left, lower-right, upper-right, and corners, respectively. We obtained this set V of vertices from the original image with a resolution of 3468 × 4624 pixels for greater accuracy in the calculation. In addition, we removed the lens distortion, obtaining the points $\left(x_{i}^{\prime },\;y_{i}^{\prime }\right)$ from the corners in screen coordinates (e.g., function *undistortPoints* of the ArUco module). In sequence, we converted each point $\left(x_{i}^{\prime },\;y_{i}^{\prime }\right),\;i\;\in \left\{1,\;2,\;3,\;4\right\}$ to the camera coordinate system by multiplying it by the inverse of the camera matrix:

$$d_{i}=\left[\begin{array}{c} x_{i}\\ y_{i}\\ 1 \end{array}\right]={\left[\begin{array}{ccc} f_{x} & 0 & c_{x}\\ 0 & f_{y} & c_{y}\\ 0 & 0 & 1 \end{array}\right]}^{-1}\left[\begin{array}{c} x_{i}^{\prime }\\ y_{i}^{\prime }\\ 1 \end{array}\right],$$

where $d_{i}\;=\;\left(x_{i},\;y_{i},\;1\right)$ indicates the 3D direction of the $v_{i}$.

**Fig. 11:**
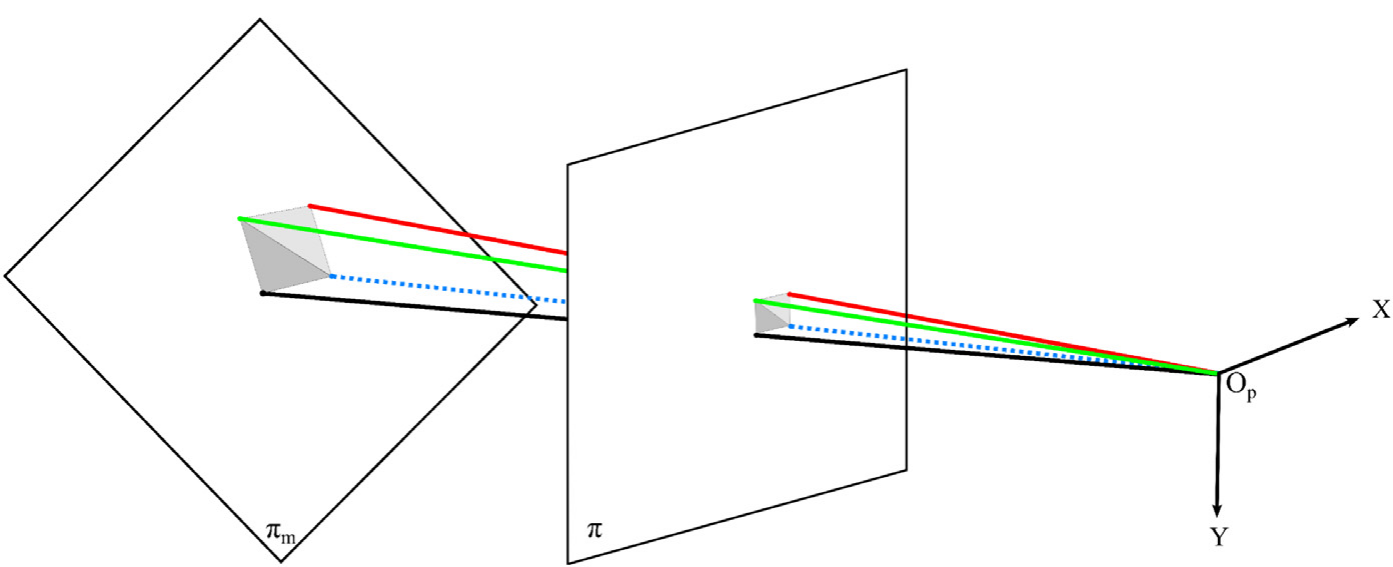
Projection of the corners of the pixel on the plane $\pi_{m}$ of the marker, passing through the plane π of image formation.

To find the plane related to the marker, we extracted the unit normal vector from the rotation matrix and used the translation vector as a point in the plane. Replacing these values in the overall equation of the plan, ax+by+cz+d=0, including the calculation of the term d, gives the equation of the marker's plane $\pi_{m}$. The intersections on $\pi_{m}$ of the lines that pass through the observer's point (0, 0, 0) at each direction $d_{i}$ of the vertices form a quadrangle on the plane $\pi_{m}$. The area of the pixel was computed by the sum of the areas of the two triangles that compose the quadrangle, as illustrated in Fig. 11.

**Fig. 12:**
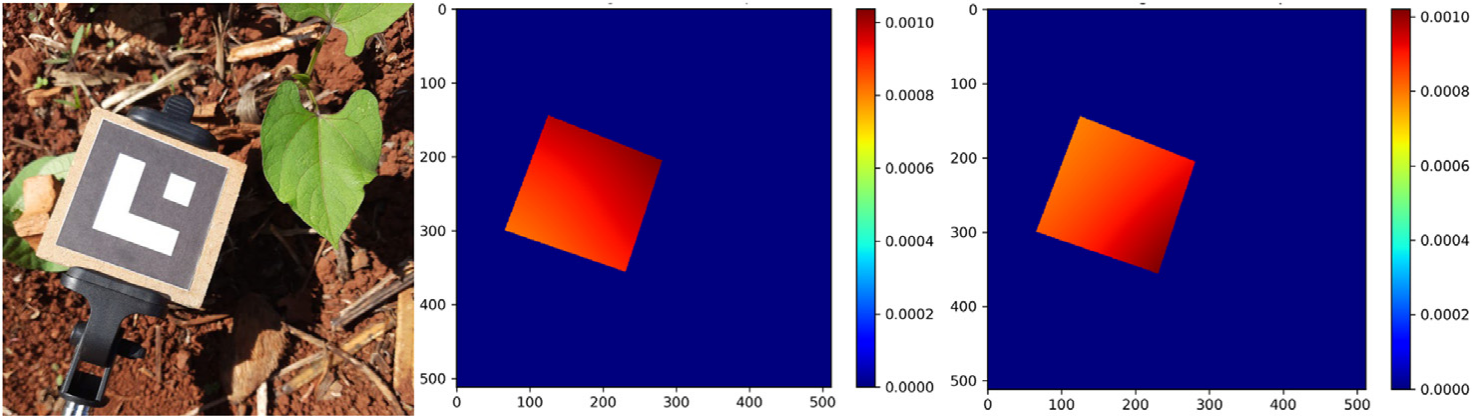
Comparison between the reduced image (left), the color map of the correct order of the corners of the marker (center) and an example of incorrect solution (right).

The pose estimation with a single image of a planar object has two possible solutions [18]. Therefore, we needed to correct the rotation and the projected area of the pixels for some markers. We have checked all possible rotations of the corners of the marker until we found the correct order, visually inspecting each solution via a color map of the projected areas, as shown in Fig. 12.

It is important to remark that the projected area may not sum up to 25 cm^2^ due to projection errors. Thus, we chose to use only the images of the markers with an absolute difference between projected area and actual area less than or equal to 1 cm^2^ to avoid distorted scenes given the estimated matrices from ArUco.

### 4.10 Cross-validation protocol definition

We propose a cross-validation protocol to establish a standard for using our dataset in training machine learning models. Our proposed protocol includes five 5-fold cross-validations. To ensure a balanced distribution and avoid bias in training, we followed the criteria:

• Images from the same plant are always included in the same fold;
• Number of leaves as similar as possible in each fold;
• Mean leaf known area as similar as possible in each fold;
• The area proportion of the leaves in each fold matches the dataset proportion (Fig. 2) as close as possible;
• Number of images as similar as possible in each fold.

Each cross-validation is composed of 5 folds following the above criteria. To accomplish this, we defined a creation procedure for each of the five cross-validations as follows:

• Procedure for Cross-validation 1: we sorted all pairs of leaves by their known area (considering the largest area of the pair) and distributed them into the five folds from the largest to the smallest area. The pair having the leaf with the greatest area is assigned to the first fold. The pair of the leaf with the second greatest area is assigned to the fold 2 and so forth;
• Procedure for Cross-validation 2: we sorted the pairs of leaves of the same plant by its mean area and distributed them into the five folds from the greater to the smaller area. The pair having the leaf with the greatest mean area is assigned to the first fold. The pair with the second greatest mean area is assigned to the fold 2 and so forth;
• Procedure for Cross-validation 3: we randomly redistributed the leaves of the Cross-validation 1 among the five folds;
• Procedure for Cross-validation 4: we randomly redistributed the leaves of the Cross-validation 2 among the five folds;
• Procedure for Cross-validation 5: we distributed the pair of leaves according to its original index in the dataset (pair 1 into fold 1, pair 2 into fold 2, and so on).

**Fig. 13:**
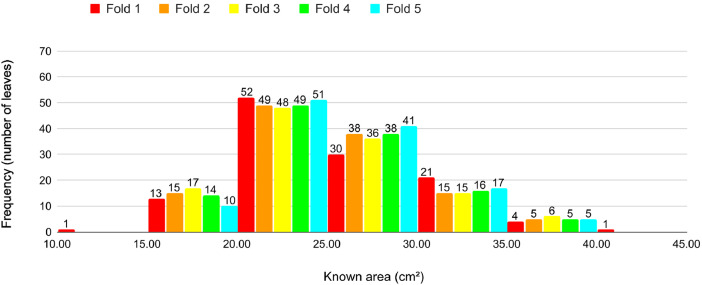
Proportion of leaves by area in each fold in Cross-validation 1.

**Fig. 14:**
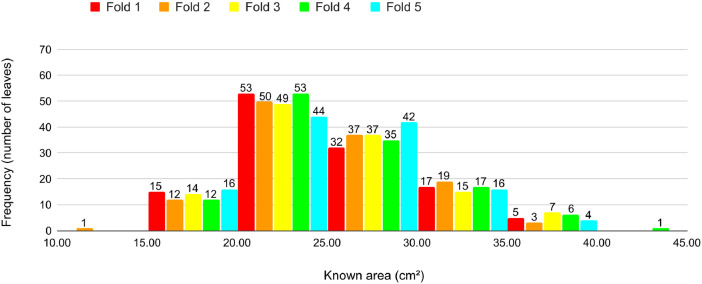
Proportion of leaves by area in each fold in Cross-validation 2.

**Fig. 15:**
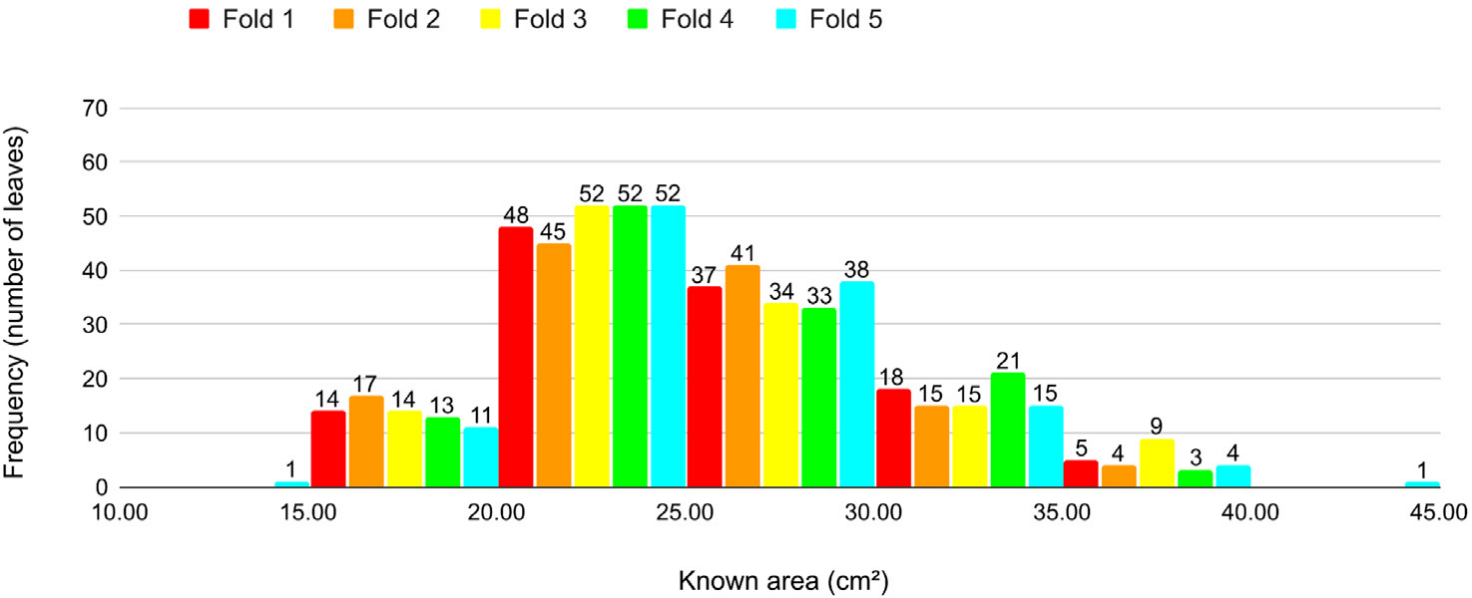
Proportion of leaves by area in each fold in Cross-validation 3.

**Fig. 16:**
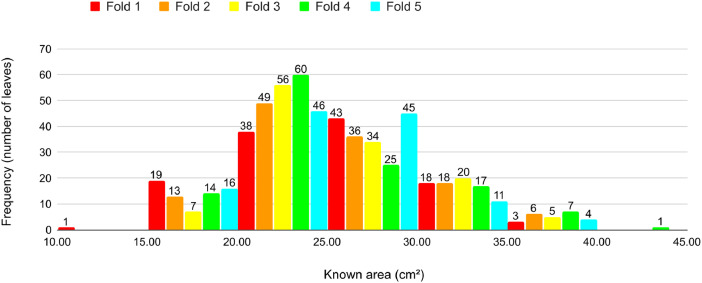
Proportion of leaves by area in each fold in Cross-validation 4.

**Fig. 17:**
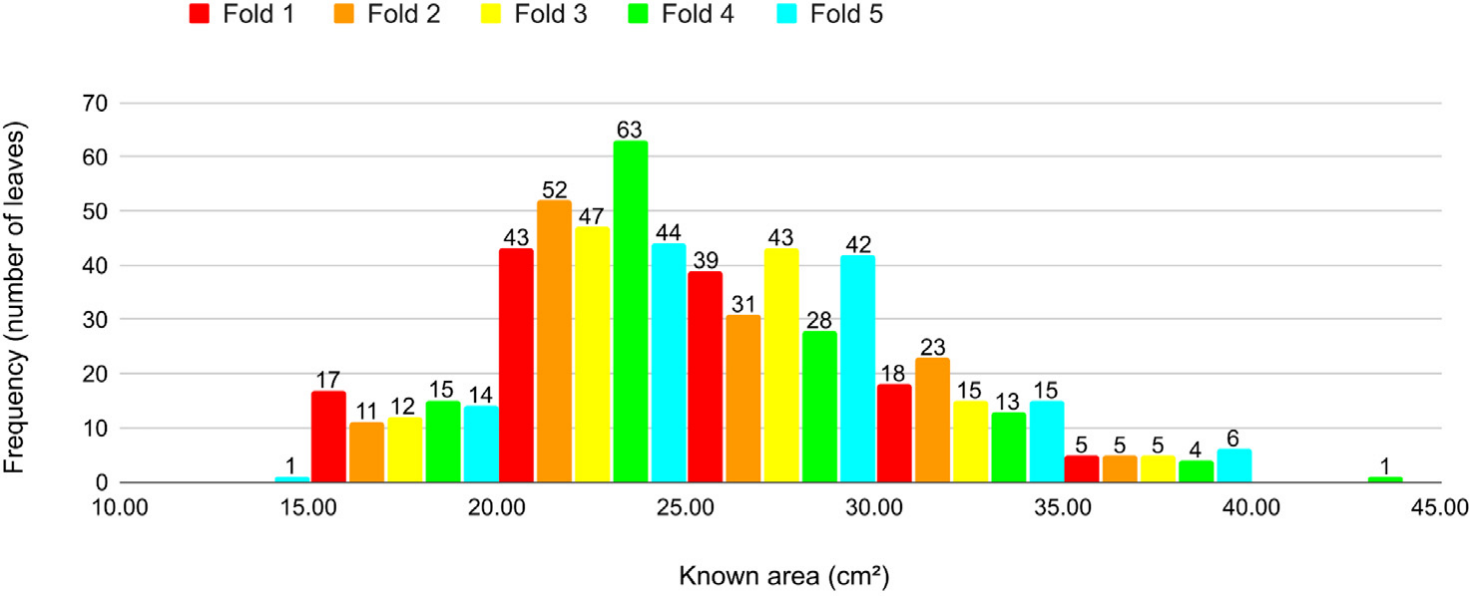
Proportion of leaves by area in each fold in Cross-validation 5.

For all procedures, some pairs of leaves were redistributed between folds trying to reinforce the criteria previously defined. Fig. 13, Fig. 14, Fig. 15, Fig. 16, Fig. 17 present the proportion of the leaves in the folds according to the area for each cross-validation. We observe a balanced proportion among the folds. Notice that Cross-validations 4 and 5 present slight proportion discrepancies in some folds. However, a high number of leaves in an area interval is compensated by a reduced number in the subsequent interval. Table 2 details the cross-validation, presenting the number of leaves, average known area and number of images per fold. The number of leaves is almost the same for every fold in each cross-validation. The average area also is similar and presents small standard deviations among the folds. The number of images presents a slightly greater variation. The greatest standard deviation is 23.8 for the Cross-validation 3 which is < 2 % of the average standard deviation of all cross-validations.

**Table 2:** Description of the proposed cross-validation protocol, including number of leaves, average known area, and number of images per fold.

Cross-validation	Fold	Number of leaves	Average known area	Number of images
1	1	122	25.363	1387
	2	122	25.361	1377
	3	122	25.351	1412
	4	122	25.374	1389
	5	124	25.480	1416
	Avg (Std)	122.4 (0.89)	25.386 (0.05)	1396.2 (15.1)
2	1	122	25.330	1406
	2	122	25.268	1358
	3	122	25.486	1395
	4	124	25.470	1417
	5	122	25.374	1405
	Avg (Std)	122.4 (0.89)	25.386 (0.08)	1396.2 (20.3)
3	1	122	25.310	1354
	2	122	25.308	1394
	3	124	25.483	1404
	4	122	25.399	1427
	5	122	25.428	1402
	Avg (Std)	122.4 (0.89)	25.386 (0.07)	1396.2 (23.8)
4	1	122	25.487	1390
	2	122	25.701	1390
	3	122	25.648	1396
	4	124	25.099	1428
	5	122	24.999	1377
	Avg (Std)	122.4 (0.89)	25.387 (0.32)	1396.2 (19.1)
5	1	122	25.368	1413
	2	122	25.607	1381
	3	122	25.549	1393
	4	124	24.933	1419
	5	122	25.480	1375
	Avg (Std)	122.4 (0.89)	25.387 (0.24)	1396.2 (17.3)

### 4.11 Utilization of the dataset for area estimation

We applied the model proposed by Silva [19], based on the DeepLabv3+ segmentation network [20], to evaluate the suitability of our dataset for the task of leaf area estimation. The neural network comprises the original encoder and decoder of the DeepLabv3+ and a new decoder specific for area estimation. We evaluated the model by using five 5-fold cross-validation with our proposed distributions. The evaluation criterion is the Relative Error Rate (RER), the percentage of the absolute difference between the estimated area performed by the model and the expected area. After training, the neural network weights of the best training step were saved and selected. The best step has the smallest sum of the average and standard deviation of the RER for the leaf and the marker. Table 3, Table 4, Table 5, Table 6, Table 7 present the results on the test set for the leaf and marker for each cross-validation. We observe that the leaf average RER for each cross-validation varies from 6.757 % for Cross-validation 5 to 8.099 % for Cross-validation 2. As expected, the model achieved a smaller RER for the marker, varying from 2.287 % to 2.714 % due to its planar geometry. The results show the applicability of our dataset for training models to estimate areas of bean leaves.

**Table 3:** Average and Standard deviation of RER for Cross-validation 1.

Fold	Leaf		Marker	
	Avg RER %	Std RER %	Avg RER %	Std RER %
1	6.933	5.805	2.475	1.479
2	7.475	5.747	1.850	1.351
3	7.491	5.635	3.170	1.520
4	7.005	5.973	3.147	1.559
5	7.207	5.796	2.098	1.392
**Average**	**7.222**	**5.791**	**2.548**	**1.460**

**Table 4:** Average and Standard deviation of RER for Cross-validation 2.

Fold	Leaf		Marker	
	Avg RER %	Std RER %	Avg RER %	Std RER %
1	8.278	7.101	2.320	1.428
2	8.324	6.623	1.919	1.347
3	8.454	6.226	2.855	1.485
4	7.745	5.896	2.645	1.568
5	7.692	6.337	1.716	1.324
**Average**	**8.099**	**6.437**	**2.291**	**1.430**

**Table 5:** Average and Standard deviation of RER for Cross-validation 3.

Fold	Leaf		Marker	
	Avg RER %	Std RER %	Avg RER %	Std RER %
1	7.865	5.994	2.458	1.436
2	8.297	6.658	3.142	1.703
3	8.004	6.473	3.342	1.599
4	6.962	5.380	2.445	1.426
5	8.108	5.717	2.183	1.421
**Average**	**7.847**	**6.044**	**2.714**	**1.517**

**Table 6:** Average and Standard deviation of RER for Cross-validation 4.

Fold	Leaf		Marker	
	Avg RER %	Std RER %	Avg RER %	Std RER %
1	7.897	6.339	3.179	1.665
2	7.296	5.355	2.579	1.467
3	6.601	5.292	1.907	1.288
4	7.398	6.134	2.046	1.406
5	6.477	5.078	2.148	1.351
**Average**	**7.134**	**5.640**	**2.372**	**1.436**

**Table 7:** Average and Standard deviation of RER for Cross-validation 5.

Fold	Leaf		Marker	
	Avg RER %	Std RER %	Avg RER %	Std RER %
1	6.636	4.927	2.014	1.409
2	6.757	5.658	2.173	1.382
3	6.275	4.942	2.665	1.561
4	7.069	5.754	2.547	1.519
5	7.048	5.938	2.035	1.303
**Average**	**6.757**	**5.444**	**2.287**	**1.435**

## Limitations

The area matrices for the leaves currently have a constant area value for all leaf points, i.e., all pixels have a constant average area which is the ratio between the leaf's total area (cm^2^) and the total number of points forming it. As a future work, we intend to complement the dataset by estimating each leaf's 3D geometry. Indeed, the leaf reconstruction allows the approximation of the true area for the leaf points.

## Ethics Statement

The authors have read and followed the ethical requirements for publication in Data in Brief and confirmed that the current work does not involve human subjects, animal experiments, or any data collected from social media platforms.

## Credit Author Statement

**Karla Gabriele Florentino da Silva:** Conceptualization, Methodology, Software, Validation, Formal analysis, Investigation, Data Curation, Writing - Original Draft, Writing - Review & Editing, Visualization. **Paulo Victor de Magalhães Rozatto:** Software, Data Curation. **Kaio de Oliveira e Sousa:** Formal analysis, Data Curation. **Lucas Dias Hudson:** Data Curation. **Artur Welerson Sott Meyer:** Data Curation. **Alemilson Fabiano Silva:** Data Curation. **Igor Tibiriçá Mendes:** Data Curation. **Alex Rodrigues Borges:** Resources, Data Curation. **Leandro Elias Morais:** Conceptualization, Resources. **Luiz Maurílio da Silva Maciel:** Conceptualization, Methodology, Validation, Resources, Writing - Original Draft, Writing - Review & Editing, Supervision, Project administration, Funding acquisition. **Saulo Moraes Villela:** Writing - Review & Editing. **Helio Pedrini:** Writing - Review & Editing. **Marcelo Bernardes Vieira:** Conceptualization, Methodology, Validation, Resources, Writing - Original Draft, Writing - Review & Editing, Supervision, Project administration, Funding acquisition.

## Data Availability

Mendeley DataLeaf on Stem Image Dataset Beans (LSID-Beans) (Original data).
